# EBUS-TBNA needle rinse fluid: a superior specimen for the molecular diagnosis of intrathoracic lymph node tuberculosis

**DOI:** 10.3389/fcimb.2025.1734956

**Published:** 2026-01-07

**Authors:** Yayan Niu, Xiaohong Li, Jin yu Yan, Huafeng Song, Lin Yao, Lijuan Zhang, Meiying Wu, Peijun Tang, Junchi Xu, Yanjun Feng

**Affiliations:** 1 Department of Tuberculosis, The Fifth People’s Hospital of Suzhou/The Affiliated Infectious Diseases Hospital, Suzhou Medical College of Soochow University, Suzhou, China; 2 Department of Traditional Chinese Medicine, The Fifth People’s Hospital of Suzhou/The Affiliated Infectious Diseases Hospital, Suzhou Medical College of Soochow University, Suzhou, China; 3 Department of Clinical Laboratory, The Fifth People’s Hospital of Suzhou/The Affiliated Infectious Diseases Hospital, Suzhou Medical College of Soochow University, Suzhou, China

**Keywords:** EBUS-TBNA, lymph node tuberculosis, molecular diagnosis, TB-DNA, Xpert MTB

## Abstract

**Objectives:**

To evaluate EBUS-TBNA needle rinse fluid versus biopsy tissue for molecular diagnosis of intrathoracic lymph node tuberculosis (TB).

**Methods:**

Retrospective analysis of 63 patients with intrathoracic lymph node TB undergoing EBUS-TBNA (2018–2024). Rinse fluid and biopsy tissue were tested via TB-DNA (n=32) and Xpert MTB/RIF (n=31); positivity rates compared.

**Results:**

The study cohort had a median age of 31 years (interquartile range: 25–50.1 years), with 57.1% (36/63) being male. The most frequently sampled lymph node stations were subcarinal (station 7, 82.5%) and right lower mediastinal (station 4R, 66.7%). Clinically, 82.5% (52/63) of patients had concomitant pulmonary TB, while 17.5% (11/63) presented with isolated intrathoracic lymph node TB. For TB-DNA detection, the positivity rate of rinse fluid (71.9%, 23/32) was significantly higher than that of biopsy tissue (46.9%, 15/32; χ²=4.146, P = 0.042). Similarly, the Xpert MTB/RIF assay showed a higher positivity rate in rinse fluid (77.4%, 24/31) compared to tissue (41.9%, 13/31; χ²=8.11, P = 0.004).

**Conclusions:**

EBUS-TBNA rinse fluid demonstrates higher sensitivity than biopsy tissue for intrathoracic lymph node TB via TB-DNA/Xpert MTB/RIF. Routine rinse fluid testing improves diagnostic yield.

## 1 Introduction

Tuberculosis (TB) is an infectious disease with high incidence and mortality rates worldwide (Chakaya et al., 2022). It is estimated that there were 10.8 million new cases of TB worldwide in 2023, with an incidence rate of 134 cases per 100,000 individuals. Although the lungs are the most common site of TB infection, TB can affect almost any part of the body (Trajman et al., 2025). Whereas pulmonary TB accounts for most of the TB cases worldwide, there has been an increasing incidence of extrapulmonary TB (EPTB) in both high- and low-income countries since the mid-1980s, with certain groups, including immunocompromised individuals and young children, exhibiting a higher frequency of EPTB (Kohli et al., 2021). EPTB accounted for 15% of the 6.3 million TB cases recorded worldwide in 2016, with the prevalence ranging from 8% in the Western Pacific region to 24% in the Eastern Mediterranean region. In England in 2019, 59.9% of TB cases were attributed to EPTB (with or without disease at another site). Additionally, 33.1% of all TB cases are categorized as TB lymphadenitis (TBLA) (Trajman et al., 2025). More than one-quarter of pulmonary TB patients also exhibit extrapulmonary manifestations (Lucey et al., 2022). The most common site of extrapulmonary TB is the lymph nodes, including the intrathoracic lymph nodes (Armstrong et al., 2019). EPTB, particularly mediastinal tuberculous lymphadenitis, presents additional diagnostic challenges relative to pulmonary TB. This result is primarily due to the need for invasive sampling procedures, which necessitate specialized expertise and, in certain instances, may involve a degree of risk to the patient. Furthermore, owing to the paucibacillary characteristics of EPTB, achieving diagnostic confirmation using the existing microbiological methods can be challenging (Chakaya et al., 2022). Patients with mediastinal lymphadenopathy require biopsy sampling to provide a pathological and/or microbiological diagnosis, particularly when this is an isolated finding (Lucey et al., 2022).

Endobronchial ultrasound-guided transbronchial needle aspiration (EBUS-TBNA) is a minimally invasive intervention that has been demonstrated to provide a superior diagnostic yield compared with conventional TBNA and shows results comparable to those of mediastinoscopy for lymph node staging in patients with primary lung cancer (Rodriguez et al., 2024). EBUS-TBNA is recommended by BTS guidelines as a safe and effective technique for the assessment of hilar and mediastinal lymph nodes in patients with suspected lung cancer and sarcoidosis (Du Rand et al., 2011). The indications for EBUS-TBNA have been expanded beyond malignant diseases to include benign diseases, such as sarcoidosis, tuberculous lymphadenitis and fungal infections (Eom et al., 2015; Berger et al., 2016). There is increasing evidence supporting the diagnostic utility and safety of EBUS-TBNA in the diagnosis of intrathoracic TBLA, particularly in patients with a high preprocedure clinical suspicion of TB (Chhajed et al., 2019).

The needle rinse fluid and biopsy tissue obtained from EBUS-TBNA can be used to detect pathogenic microorganisms, and molecular biology methods can be used to detect *Mycobacterium tuberculosis* (MTB). Consequently, this study aimed to evaluate the diagnostic utility of flushing fluid and biopsy tissue obtained through EBUS-TBNA in the identification of mediastinal lymph node TB.

## 2 Materials and methods

### 2.1 Study design

A retrospective study was conducted of suspected mediastinal or hilar lymph node TB patients who received treatment at Suzhou Fifth People’s Hospital from February 2018 to July 2024. All the subjects underwent EBUS-TBNA. The inclusion criteria were as follows: (1) patients whose mediastinal lymph nodes and/or hilar lymph nodes had a maximum short diameter greater than 1.0 cm on enhanced CT; (2) patients who demonstrated tolerance to EBUS-TBNA; and (3) patients whose legal guardians provided signed informed consent for bronchoscopic examination. Both TB-DNA and Xpert MTB/RIF assays were concurrently conducted on specimens obtained from EBUS-TBNA wash fluid or biopsy samples. The exclusion criteria included (1) pregnant or lactating individual; (2) HIV-positive individuals; (3) individuals with uncontrolled hypertension or severe cardiovascular or cerebrovascular diseases; (4) individuals with coagulation dysfunction or bleeding diathesis; and (5) individuals who experienced cerebral infarction or cerebral hemorrhage within the previous 3 months. Following comprehensive evaluation, a total of 63 patients were ultimately enrolled and diagnosed with mediastinal or hilar lymph node TB.

The IRB waived the requirement for informed consent because of the retrospective nature of the study. All patient records and data were anonymized and deidentified prior to analysis.

### 2.2 Study population and sample collection

A dedicated, qualified, and experienced bronchoscopist performed the procedures. The target lymph nodes were selected by a pulmonologist on the basis of imaging results, including chest computed tomography (CT) and positron emission tomography (PET)-CT results if available. Subsequently, bronchoscopy was conducted. Physicochemical inhalation of lidocaine hydrochloride was used for pharyngeal and laryngeal anesthesia. First, the patients underwent general bronchoscopy (Olympus Corporation, Japan; #BF-UC260FW) to evaluate the lumen of each segment of the trachea and bronchus. Afterward, endobronchial ultrasound (EBUS) (Olympus Corporation, Japan, #EU-ME2) bronchoscopy was performed. Target lymph nodes were sequentially identified using ultrasound imaging, with measurements obtained of their size and axial depth. Upon determining the puncture site, a specialized needle (Olympus Corporation, Japan, # NA-201SX-4022) was introduced through the working channel of the bronchoscope. Lymph node puncture was performed under real-time ultrasound guidance. The collected samples were placed in a centrifuge tube, and the puncture needle was flushed with 1 ml of sterile normal saline to obtain a sample of the needle rinse fluid. The tissue samples in the centrifuge tube were subsequently fixed in formaldehyde and subjected to histopathological examination, as well as TB-DNA (Boao Bio Co., Ltd.) and/or Xpert MTB/RIF (Cepheid, USA) testing, depending on the number of samples obtained. All rinse fluid samples were collected in an aseptic tube and sent for TB-DNA and Xpert MTB/RIF and culture assays.

### 2.3 Procedure

To collect and analyze clinical data from 134 patients who underwent EBUS-TBNA for lymph node tissue sampling, with specimens obtained both before and after testing for TB-DNA, the Xpert MTB/RIF assay, and histopathological examination were conducted. To examine the clinical characteristics of patients diagnosed with intrathoracic lymph node TB. To compare the diagnostic sensitivity levels of TB-DNA and Xpert MTB/RIF assays individually in detecting MTB in EBUS-TBNA needle rinse fluid and tissue samples.

### 2.4 Statistics

Statistical analyses were conducted using SPSS (version 25.0). Continuous variables that followed a normal distribution are presented as the mean ± standard deviation (X ± s), whereas those not conforming to a normal distribution are expressed as the median (Q1, Q3). Comparisons between groups for continuous variables were performed using the independent samples t test. Categorical data are reported as frequencies and percentages (%), and group comparisons were conducted using the chi-square test or Fisher’s exact test, as appropriate.

## 3 Results

### 3.1 Baseline

**Figure 1:**
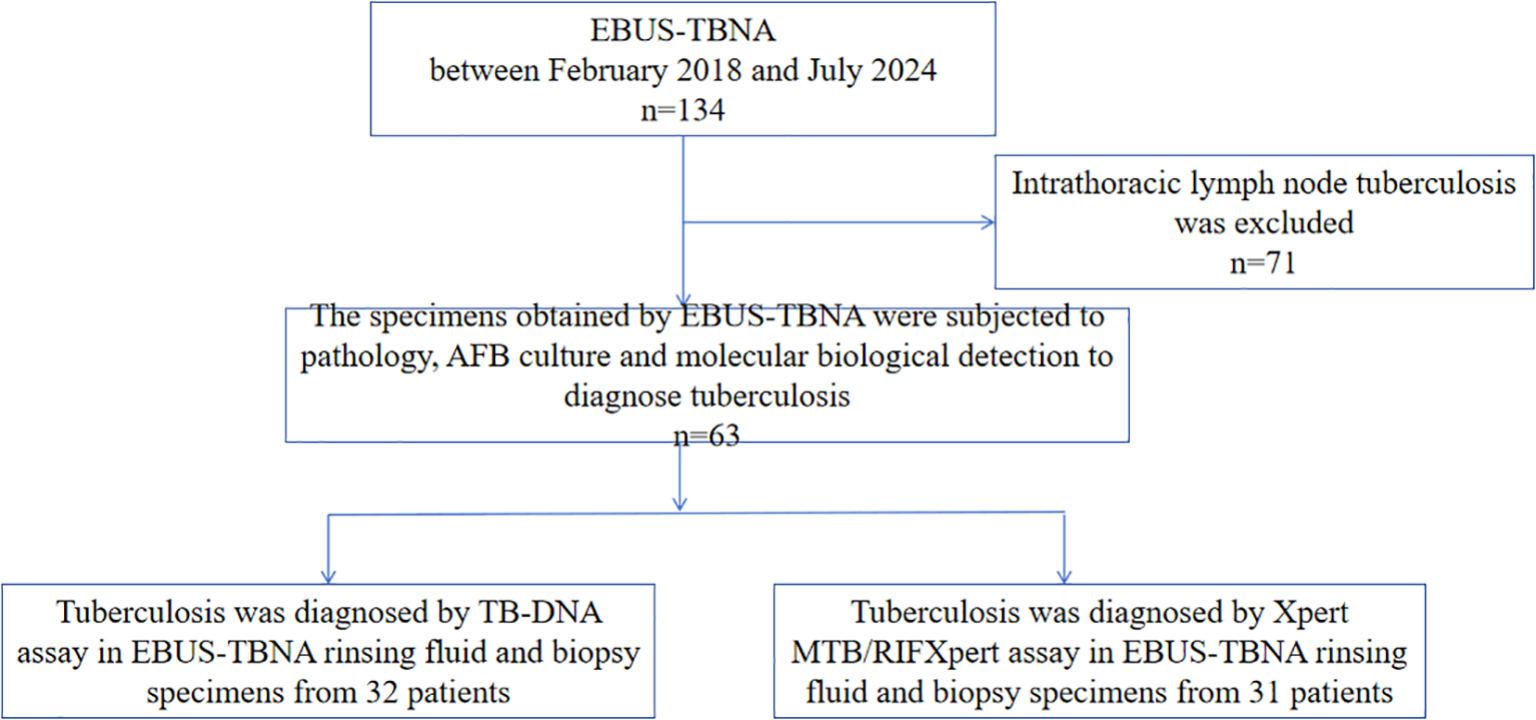
Flow chart of the study. AFB, acid-fast bacilli; EBUS-TBNA, endobronchial ultrasound-guided transbronchial needle aspiration.

From February 2018 to July 2024, a total of 134 EBUS-TBNAs were conducted on the basis of radiological indications of intrathoracic lymphadenopathy. Among the patients who underwent EBUS-TBNA, 71 (53%) were diagnosed with non-TB, while 63 (47%) were confirmed to have TB. Among the cohort, 32 patients had their EBUS-TBNA needle rinse fluid and biopsy specimens analyzed for TB DNA (TB-DNA), and 31 patients underwent testing of their EBUS-TBNA needle rinse fluid and biopsy specimens using the Xpert MTB/RIF assay (**Figure 1**).

The baseline characteristics of patients diagnosed with intrathoracic lymph node TB are presented in **Table 1**. The median patient age was 31 years (IQR, 25–50.1), and 36 (57.1%) were male. Twenty-one patients (11%) had comorbidities, with autoimmune disease being the most common. Five patients (7.9%) had a history of previous anti-TB treatment. At the time of diagnosis, 50 patients (79.4%) had thoracic symptoms, including cough and sputum, and 43 patients (68.3%) had constitutional symptoms such as sweating or debilitation. The mean white blood cell, neutrophil, and lymphocyte counts were 6.3/μL, 4.1/μL, and 1.4/μL, respectively. The mean values of CRP and ERS were 20.6 mg/dL and 29.8 mm/hra, respectively. The mean values of hemoglobin and albumin were 128.5 mg/dL and 39.5 mm/hra, respectively.

**Table 1:** Baseline characteristics of patients with intrathoracic lymph nodes TB (n = 63).

Variables	Values (n = 63)
Sex, male (%)	36 (57.1)
Age, yr	31 (25-50.1)
Body mass index, kg/m^2^	20.8 (19.7-23.5)
Comorbidities (%)	21 (33.3)
Diabetes mellitus (%)	4 (6.3)
Hypertension (%)	3 (4.7)
Autoimmune disease (%)	7 (11.1)
Chronic pulmonary disease (%)	2 (3.2)
Chronic kidney disease (%)	2 (3.2)
Chronic liver disease (%)	2 (3.2)
Malignancy (%)	2 (3.2)
Previous history of anti-TB treatment (%)	5 (7.9)
Symptoms	
Thoracic symptom (%)	50 (79.4)
Cough (%)	45 (71.4)
Sputum (%)	32 (50.7)
Hemoptysis (%)	4 (4.7)
Dyspnea (%)	10 (15.8)
Chest pain (%)	10 (15.8)
Dysphagia (%)	4 (4.7)
Constitutional symptom (%)	43 (68.3)
Fever (%)	19 (30.1)
Sweating (%)	31 (49.2)
Debilitation (%)	30 (47.6)
Weight loss (%)	16 (25.3)
Asitia (%)	8 (12.6)
Laboratory findings	
White blood cell,/µL	6.3 ± 2.1
Neutrophils,/µL	4.1 ± 1.8
Platelet,/µL	244.4 ± 91.1
Lymphocytes,/µL	1.4 ± 0.6
CRP, mg/dL	20.6 ± 32.4
ESR, mm/hra	29.8 ± 27.8
Hemoglobin, g/L	128.5 ± 18.9
Albumin, g/L	39.5 ± 4.3

All variables are values at the time of diagnosis of intrathoracic lymph nodes TB. CRP, C-reactive protein; ESR, Erythrocyte sedimentation rate.

### 3.2 Distribution of mediastinal and hilar lymph node locations

The distribution of sampled lymph node stations across the 63 patients was presented in **Supplementary Table 1**. A total of 156 lymph nodes were sampled. Among these, the subcarinal (station 7, 82.5%) and the right lower paratracheal (station 4R, 66.7%) stations were the most frequently sampled. The number of enlarged lymph nodes in the mediastinal lymph nodes was significantly greater than that in the hilar region. Most patients had hilar lymph node enlargement (**Table 2**).

**Table 2:** The site and sampled lymph node stations. (n = 63).

Station	N (%)
2R	18 (28.6)
4R	42 (66.7)
4L	8 (12.7)
7	52 (82.5)
10R	1 (1.6)
11Rs	19 (30.2)
11Ri	5 (7.9)
11L	9 (14.3)
12R	2 (3.2)
Site of lymph nodes	N (%)
Only mediastinal	30 (47.6)
Hilar + mediastinal	23 (36.5)
Only hilar	2 (3.2)

### 3.3 Clinical features of intrathoracic lymph node TB with or without pulmonary TB

Among the 63 patients diagnosed with intrathoracic lymph node TB, only 11 patients had isolated intrathoracic lymph node TB, while 52 patients exhibited concomitant pulmonary TB. Comparative analysis revealed that respiratory symptoms differed significantly between patients with isolated intrathoracic lymph node TB and patients with combined intrathoracic lymph node and pulmonary TB. However, no significant differences were observed between the two groups in terms of TB-related systemic symptoms, inflammatory markers, nutritional status, body mass index (BMI), white blood cell counts, neutrophil and lymphocyte counts, or lymphocyte subset distribution (**Table 3**).

**Table 3:** Comparison of clinical features of intrathoracic lymph node TB with or without pulmonary TB (n = 63).

Characteristics	Without PTB (n=11)	With PTB (n=52)	Statistical value	P value
Male (%)	5 (45.5)	31 (49.2)	0.278	0.598
Age, yr	28 (24.5-32.5)	31 (25-52.3)	-1.170	0.242
Body mass index, kg/m^2^	21.9 (20.9-25.7)	20.8 (19.5-22.7)	-1.266	0.206
Thoracic symptom (%)	3 (27.3)	47 (74.6)	18.497	0.000
Constitutional symptom (%)	6 (54.5)	37 (58.7)	0.516	0.472
CRP, mg/dL	6.4 (4.7-11.5)	7.5 (3.4-26.6)	-0.562	0.574
ESR, mm/hra	24 (11.8-36.5)	25.5 (12.3-37.5)	-0.107	0.915
Hemoglobin	131.7 ± 4.8	127.8 ± 2.8	0.616	0.540
Albumin	40.9 ± 0.8	39.2 ± 0.6	1.159	0.251
White blood cell,/µL	6.1 ± 0.6	6.3 ± 0.3	-0.270	0.799
Neutrophils,/µL	3.8 ± 0.6	4.2 ± 03	-0.609	0.545
Platelet	231.8 ± 26.7	247.1 ± 12.9	-0.498	0.620
Lymphocytes,/µL	1.7 ± 0.2	1.4 ± 0.1	1.578	0.120
CD4+T cell (%)	39.8 ± 3.7	38.7 ± 1.5	0.292	0.772
CD8+T cell (%)	23.4 ± 2.1	27 ± 1.3	-1.201	0.237
B cell (%)	14.2 ± 2.4	14 ± 1.2	0.063	0.950
NK cell (%)	14.7 ± 3.8	13.7 ± 1.5	0.264	0.793

**Figure 2:**
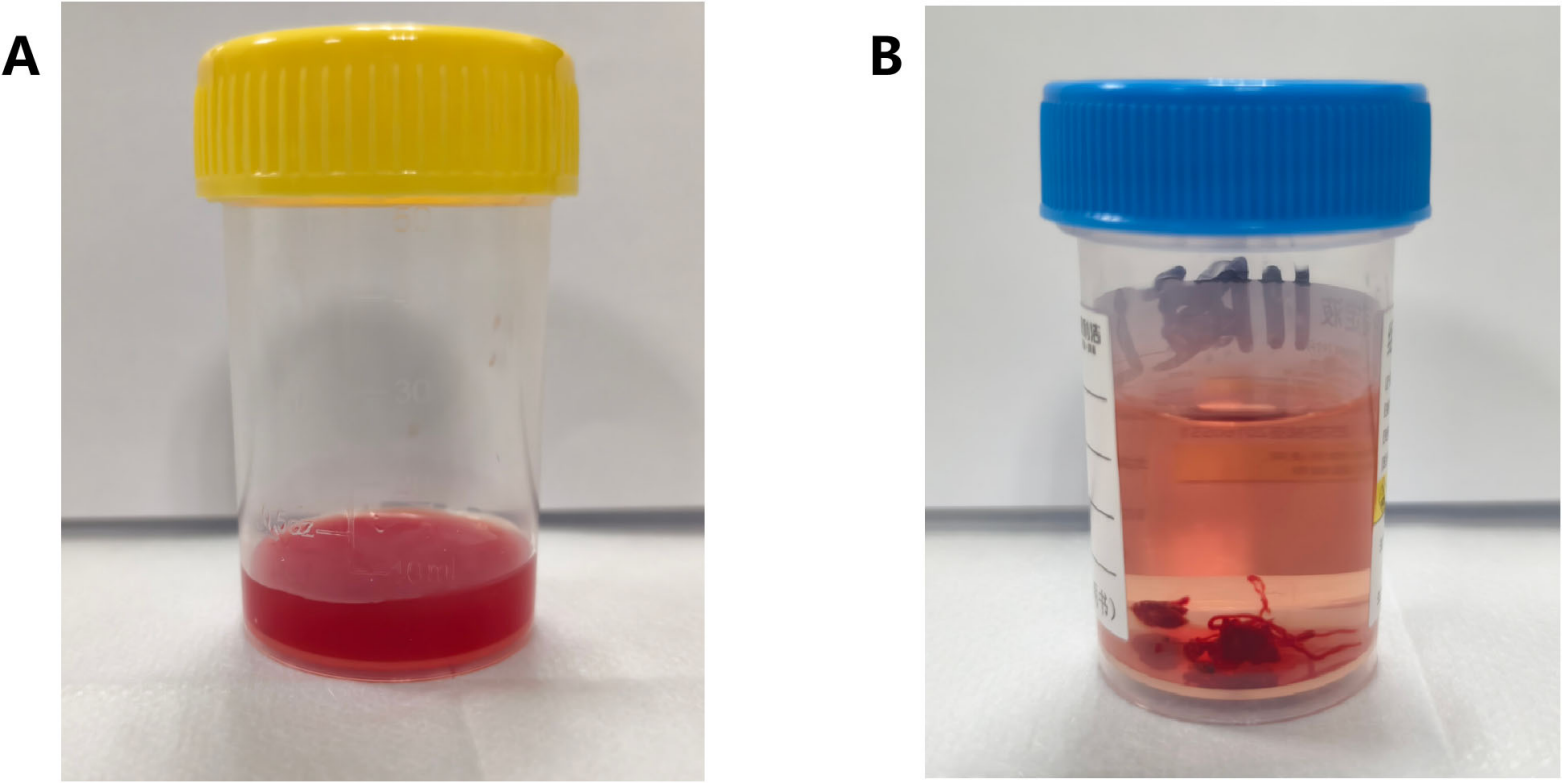
The EBUS needle rinse fluid **(A)** and EBUS TBNA biopsy tissue **(B)** were used to detect TB by TB-DNA or Xpert MTB/RIF. A:EBUS needle rinse fluid; B:EBUS TBNA biopsy tissue.

The EBUS needle rinse fluid (**Figure 2A**) and EBUS TBNA biopsy tissue (**Figure 2B**) were used to detect tuberculosis by TB-DNA or Xpert MTB/RIF. Among the 63 patients diagnosed with intrathoracic lymph node TB, 32 underwent TB-DNA testing on both the needle rinse fluid and the biopsy tissue, while 31 underwent Xpert MTB/RIF testing on both sample types. The positivity rate of TB-DNA in the needle rinse fluid was 71.9%, whereas it was 46.9% in the biopsy tissue (**Table 4**). Furthermore, the Xpert MTB/RIF assay demonstrated a positive rate of 77.4% in the needle rinse fluid, whereas the positivity rate in the biopsy tissue was 41.9% (**Table 5**).

**Table 4:** The TB-DNA method was used to diagnose puncture EBUS needle rinse fluid and EBUS TBNA biopsy tissue (n = 32).

Sample	Positive	Negative	Positive rate	Statistical value	P value
Needle rinse fluid	23	9	71.9%	χ^2^ = 4.146	0.042
Biopsy tissue	15	17	46.9%		

**Table 5:** The Xpert MTB/RIF method was used to diagnose puncture EBUS needle rinse fluid and EBUS TBNA biopsy tissue (n = 31).

Sample	Positive	Negative	Positive rate	Statistical value	P value
Needle rinse fluid	24	7	77.4%	χ^2^ = 8.11	0.004
Biopsy tissue	13	18	41.9%		

Among the 63 intrathoracic lymph node TB patients, 51 underwent puncture EBUS needle rinse fluid testing. Of these, 47 tested positive and 4 tested negative. Their clinical characteristics are presented in **Supplementary Table 2**. What’s more, among the 63 patients, 47 were diagnosed with tuberculosis using needle rinse fluid, 40 were diagnosed with biopsy tissue samples, and 24 were diagnosed using both biopsy tissue and needle rinse fluid. Using the Xpert MTB/RIF method, 59 cases were diagnosed with TB, while the TB-DNA method diagnosed 42 cases; 38 cases were diagnosed by both methods simultaneously (**Supplementary Table 3**).

## 4 Discussion

The primary aim of this study was to evaluate the diagnostic significance of EBUS-TBNA fluid in detecting intrathoracic lymph node TB. The patient cohort mainly comprised young males with minimal comorbidities, indicating no clear demographic predisposition for the development of intrathoracic lymph node TB. Previous literature has reported that mediastinal lymph node TB represents a common form of TB, accounting for approximately 8% to 10% of TB cases (Baran et al., 1996). Consistent with these findings, this study revealed that intrathoracic lymph node TB frequently co-occurred with pulmonary TB, and isolated cases of intrathoracic lymph node TB were relatively infrequent.

Intrathoracic lymph node TB lacks distinctive clinical symptoms, and abnormal chest CT images may be its unique manifestation; it often simulates malignancy or other inflammatory diseases (Navani et al., 2011; Nin et al., 2016). In this study, differences were observed only in respiratory symptoms between patients with isolated intrathoracic lymph node TB and those with pulmonary TB. No significant differences were found in TB-related systemic symptoms, inflammatory markers, nutritional status, white blood cell counts, neutrophil and lymphocyte levels, or lymphocyte subsets. These findings suggest that the clinical presentation of intrathoracic lymph node TB is nonspecific, corroborating the findings of previous reports.

Owing to its hidden location, the diagnosis of intrathoracic tuberculous lymphadenitis is usually challenging because of the difficulty of the approach and the lack of specific clinical and radiological characteristics. The acquisition of intrathoracic lymph node specimens and the use of sensitive MTB detection methods are two primary obstacles to diagnosis. Ye et al. A meta-analysis of the diagnostic efficacy of EBUS-TBNA in intrathoracic LNTB revealed a pooled sensitivity of 0.80 (95% CI, 0.74–0.85) and a specificity of 1.0 (95% CI, 0.99–1.00) on the basis of 7 studies with sufficient data for these calculations. Another London-based study by Navani et al. reported that the sensitivity of EBUS-TBNA was 94% (95% CI, 0.88–0.97). Olivia Lucey et al. confirmed the utility of EBUS-TBNA in the diagnosis of intrathoracic TB in an undifferentiated cohort of patients with mediastinal lymphadenopathy of unknown etiology (Sun et al., 2021).

Although a consensus among experts has established that the identification of characteristic pathological lesions, namely, chronic granulomatous inflammation with caseous necrosis, via histopathological examination serves as a diagnostic marker for lymph node TB (Sun et al., 2021), in the absence of direct evidence of MTB and when only nonspecific pathological features such as inflammatory exudation, cellular proliferation, or necrosis are present, histopathology often yields suggestive rather than definitive diagnoses (Xu et al., 2024) Given that TB is an infectious disease, etiological confirmation remains fundamental for diagnosing intrathoracic lymph node TB. Culture-based strain identification and drug susceptibility testing are regarded as the diagnostic gold standards; however, these procedures may take several weeks to months to yield results (Demers et al., 2016). Consequently, early and rapid diagnostic methods are urgently needed. Molecular biological techniques for detecting MTB fulfill this requirement. The Xpert MTB/RIF assay, a molecular diagnostic tool endorsed by the World Health Organization (WHO), enables the detection of MTB and rifampicin resistance in pulmonary specimens (e.g., sputum) within approximately two hours (Haridas et al., 2022). Furthermore, the WHO recommends the application of Xpert MTB/RIF for nonpulmonary specimens, such as cerebrospinal fluid and lymph node samples, in cases suspected of extrapulmonary TB (Maynard-Smith et al., 2014). The MTB nucleic acid (TB-DNA) detection kit, developed in China, relies on the use of dual real-time polymerase chain reaction (PCR) technology combined with TaqMan probe technology to detect mycobacterial nucleic acids, completing analysis within three hours and facilitating rapid diagnosis. This PCR-based assay has received certification from the Chinese Food and Drug Administration and enables prompt detection of MTB nucleic acid. Its diagnostic performance is comparable to that of acid-fast bacilli detection in sputum smears, assisting in the diagnosis of smear-positive pulmonary TB and enhancing diagnostic accuracy in smear-negative cases. It represents a rapid and sensitive method for pathogen identification (Cheng et al., 2024). A systematic review and meta-analysis demonstrated that the Xpert MTB/RIF assay has high diagnostic accuracy for detecting lymph node aspirates, although the majority of the included studies focused on peripheral lymph nodes (Kohli et al., 2021). Another study within this meta-analysis evaluated the diagnostic performance of Xpert MTB/RIF on samples obtained via EBUS-TBNA and concluded that this technology has satisfactory sensitivity for diagnosing mediastinal tuberculous lymphadenitis (Mondoni et al., 2023).

A total of 156 lymph node stations were sampled from 63 patients. The total number of sampled stations exceeded the number of patients because multiple stations (ranging from 1 to 5) were punctured per patient. The most frequently sampled stations were the subcarinal (station 7; 82.5%) and the right lower paratracheal (station 4R; 66.7%). Given that the majority of patients undergo EBUS-TBNA for cancer evaluation, it is critical to acquire an adequate quantity of core tissue to facilitate comprehensive histopathological analysis and molecular diagnostics. Specimens obtained via EBUS-TBNA can be subjected to diverse analytical techniques, including cytological assessment, flow cytometry, and cell block preparation for immunohistochemical or molecular investigations, as well as microbiological evaluations such as culture (Ko et al., 2020). Prior research focusing on TB mainly involve the use of biopsy samples acquired through EBUS-TBNA for the detection of MTB, with limited studies employing EBUS-TBNA needle rinse fluid for this purpose (Ko et al., 2019). The EBUS-TBNA needle rinse fluid, obtained by irrigating the needle lumen with sterile normal saline after core tissue retrieval, represents an additional sample that can be rapidly collected within seconds. Investigations have demonstrated that the MTB burden in EBUS-TBNA flushing fluid is relatively low; specifically, only 1.9% of patients with intrathoracic TB lymphadenitis yielded positive smear results from this fluid. Nonetheless, routine mycobacterial culture of EBUS-TBNA needle flushing fluid has proven effective for TB detection and monitoring [30]. Given the comparable sensitivities of the Xpert MTB/RIF assay and mycobacterial culture, it is reasonable to infer that molecular diagnostic modalities such as Xpert MTB/RIF are well suited for detecting MTB in EBUS-TBNA needle flushing specimens.

Despite the retrospective nature of this study, our findings indicate that both the Xpert MTB/RIF assay and the TB-DNA method yielded significantly higher positivity rates in EBUS-TBNA flushing fluid than in EBUS-TBNA biopsy tissue samples. These results underscore the considerable diagnostic value of EBUS-TBNA flushing fluid in detecting intrathoracic lymph node TB. Furthermore, the analysis of flushing fluid offers additional benefits, as it does not necessitate extra puncture procedures or increased operation time.

However, retrospective design inherently suffers from potential selection and information bias. Since the diagnostic value of needle rinse fluid for TB remains unclear, not all patients underwent concurrent collection of biopsy specimens and rinse fluid for testing. In clinical practice, due to considerations of patients’ medical costs, needle rinse fluid was not collected for tuberculosis testing in patients with non-TB lymph node disease. In addition, this study has several other limitations. First, the retrospective design may introduce selection bias, potentially influencing the findings. Therefore, future prospective cohort studies are warranted to validate these findings. Second, among the 63 patients diagnosed with intrathoracic lymph node TB, not all underwent collection of both EBUS-TBNA flushing fluid and tissue samples for Xpert MTB/RIF and TB-DNA testing, primarily owing to sample availability and financial constraints, resulting in a reduced sample size for analysis. Although this study had a relatively small sample size, a statistically significant difference in the positive rate was observed in the Xpert MTB/RIF group (P = 0.004), suggesting a substantial effect size and a certain degree of reliability in the findings. Third, the limited number of MTB cultures performed, which is attributable to sample quantity and economic factors, precluded the analysis of culture results from the EBUS-TBNA flushing fluid and tissue specimens.

## 5 Conclusions

In summary, we recommend that EBUS-TBNA flushing fluid should be used as a diagnostic specimen for intrathoracic lymph node TB. Furthermore, when resources permit, simultaneous collection of both EBUS-TBNA flushing fluid and tissue samples for MTB detection is recommended to increase diagnostic accuracy. 

## Data Availability

The original contributions presented in the study are included in the article/**Supplementary Material**. Further inquiries can be directed to the corresponding authors.
