# Supplementary material for: EBUS-TBNA needle rinse fluid: a superior specimen for the molecular diagnosis of intrathoracic lymph node tuberculosis
Source: Front Cell Infect Microbiol. 2026 Jan 7;15:1734956. doi: 10.3389/fcimb.2025.1734956 (PMC12819615; doi:10.3389/fcimb.2025.1734956)
Supplement: Supplementary file 1 [file Table1.docx]

**Supplement Table 1. The distribution of 156 punctured lymph node stations from the 63 patients.**

| Stations | patients (n) |
| --- | --- |
| 7 | 9 |
| 4R, 7 | 8 |
| 2R, 4R, 7 | 6 |
| 4R | 3 |
| 2R, 4R | 3 |
| 4L, 4R, 7 | 2 |
| 2R, 7 | 2 |
| 4R, 7, 11R | 2 |
| 4R, 7, 11L, 11RS | 2 |
| 2R, 4R, 11RS, 11L | 1 |
| 2R | 1 |
| 11R, 7 | 1 |
| 4L, 4R, 7, 11L | 1 |
| 4R, 4L, 7 | 1 |
| 4R, 11Rs | 1 |
| 2R, 4R, 4L, 7 | 1 |
| 4L, 11L, 7 | 1 |
| 2R, 4R, 7, 11R, 11L | 1 |
| 7, 11R | 1 |
| 7, 11L | 1 |
| 11L, 7 | 1 |
| 12R, 11R, 7 | 1 |
| 2R, 4R, 7, 11R | 1 |
| 2R, 4R, 7, 10R | 1 |
| 2R, 11R, 7 | 1 |
| 4L, 4R, 7, 11 | 1 |
| 7, 11R, 11L | 1 |
| 4L, 4R, 7, 11R | 1 |
| 4R, 7, 11R, 12R, 11L | 1 |
| 2R, 4R, 11R | 1 |
| 4R, 7, 11Rs | 1 |
| 2R, 4R, 7, 10R, 11Rs | 1 |
| 10R, 11L | 1 |
| 4R, 7, 11Rs, 11L | 1 |
| 4R, 7, 11Rs, 11Ri | 1 |
|  |  |

**Supplement Table 2. Clinical characteristics of the 51 patients undergoing EBUS needle rinse fluid testing.**

| Variables | Needle rinse  fluid positive (n=47) | Needle rinse  fluid negative (n=4) | Statistical value | P value |
| --- | --- | --- | --- | --- |
| Male (%) | 23 (48.9) | 3 (75) | 0.23 | 0.631 |
| Age, yr | 40.5±17.7 | 24.3±7.8 | 3.459 | 0.013 |
| Body mass index, kg/m2 | 21.4±3.5 | 22.7±2.7 | -0.59 | 0.558 |
| Thoracic symptom (%) | 37 (78.7) | 3 (75) | 1 | 0.634 |
| Constitutional symptom (%) | 27 (57.4) | 2 (50) | 1 | 0.664 |
| Complications of pulmonary TB | 41 (87.2) | 3 (75) | 0.457 | 0.457 |
| Complications of extrapulmonary TB | 14 (29.8) | 1 (25) | 1 | 0.664 |
| CRP, mg/dL | 16.7±22.3 | 28.1±33.9 | -0.716 | 0.522 |
| ESR, mm/hra | 32.9±33.0 | 20.3±14.8 | -0.554 | 0.58 |
| Hemoglobin | 127.5±18.8 | 130.8±30.2 | -0.333 | 0.739 |
| Albumin | 39.8±4.1 | 40.5±3.9 | -0.053 | 0.958 |
| White blood cell, /µL | 6.0±2.1 | 7.5±3.2 | -0.946 | 0.344 |
| Neutrophils, /µL | 3.9±1.8 | 5.0±3.0 | -0.666 | 0.506 |
| Plateletl, /µL | 238.3±94.6 | 253.5±105.7 | -0.07 | 0.944 |
| Lymphocytes, /µL | 1.4±0.6 | 1.6±0.4 | -1.034 | 0.301 |
| CD4+T cell (%) | 39.4±9.1 | 36.7±7.7 | -0.53 | 0.596 |
| CD8+T cell (%) | 27.7±7.9 | 25.6±6.8 | -0.412 | 0.68 |
| B cell (%) | 13.2±6.4 | 20.7±2.5 | -2.2121 | 0.034 |
| NK cell (%) | 13.3±8.8 | 10.1±7.3 | -0.648 | 0.517 |

**Supplement Table 3. TB diagnosis positive rate for rinse fluid or biopsy tissue, and Xpert MTB/RIF assay or TB-DNA method.**

| Variables | TB diagnosis positive N (%) |
| --- | --- |
| Sample types |  |
| Needle rinse fluid | 47 (74.6%) |
| Biopsy tissue | 40 (63.5%) |
| Both | 24 (38.1%) |
| Molecular methods |  |
| Xpert MTB/RIF | 59 (93.7%) |
| TB-DNA | 42 (66.7%) |
| Both | 38 (60.3%) |
